# A Phase 3 Clinical Protocol: Placebo‐Controlled, Double‐Blind, Parallel‐Group to Study the Safety and Efficacy of NA‐831 in Combination with Donanemab in Subjects with Early Alzheimer's Disease

**DOI:** 10.1002/alz70861_108266

**Published:** 2025-12-23

**Authors:** Lloyd Tran, Fern Vu, Zung Vu Tran

**Affiliations:** ^1^ Biomed Industries, Inc., San Jose, CA USA; ^2^ MedAware Systems, Inc., Broomfield, CO USA

## Abstract

**Background:**

Donanemab has recently been approved by the FDA for patients with early Alzheimer’s disease (AD). NA‐831 is an investigational drug that demonstrated proof of safety and efficacy in a Phase 2 clinical trial for patients with early AD. Patients with AD are often treated with comedications to address both cognitive impairment and related comorbidities. This study aims to evaluate the efficacy and safety of combination therapy with NA‐831 and donanemab in early Alzheimer’s disease (EAD).

**Method:**

The phase 3 study consists of a Core and Open Label Extension (OLE) Phase will be conducted to evaluate the efficacy and safety of combination therapy of NA‐ 831 and donanemab with patients with EAD. The Core is an 18‐month treatment, multi‐centers, double blind, placebo controlled parallel group study.

Enrollment: 600 participants. Ages: 50 to 90 Years. All sexes are eligible for study.

**Key Inclusion Criteria:**

• Meet the NIA‐AA core clinical criteria for probable Alzheimer's disease dementia

• Have a global CDR score of 0.5 to 1.0 and a CDR Memory Box score of 0.5 or greater at Screening and Baseline.

• Mini mental state examination (MMSE) score >=22 at Screening and Baseline and <=30 at Screening and Baseline

**Core Study:**

Participants will be divided in 3 groups:

Group 1: will receive one 30 mg of NA‐831 capsule orally once a day or placebo.

Group 2: will receive and intravenous donanemab (700 mg every 4 weeks) or placebo.

Group 3: will receive one 30 mg of NA‐831 capsule orally once a day and intravenous donanemab (350 mg every 4 weeks) or placebo.

Open Label Extension Phase: Participants completing the core study will receive one 30 mg of NA‐31 capsule orally once a day, and intravenous donanemab (350 mg every 2 weeks)

**Result:**

The Phase 3 trial is underway across more than 30 sites in the United States and internationally. Detailed methodology and protocol will be presented and discussed.

**Conclusion:**

Final results from the Phase 3 study will be announced upon completion. The findings are expected to provide valuable insights into the safety and efficacy of combination therapy with NA‐831 and donanemab for early Alzheimer’s disease.

